# Water determines the intramolecular dynamics of proteins. En example of bovine serum albumin

**DOI:** 10.3389/fchem.2024.1444448

**Published:** 2024-07-25

**Authors:** Nikita V. Penkov

**Affiliations:** Institute of Cell Biophysics, Federal Research Center “Pushchino Scientific Center for Biological Research of the Russian Academy of Sciences”, Pushchino, Russia

**Keywords:** protein dynamics, dielectric spectra, THz, THz-TDS, hydrate shells, native conformation, BSA

## Abstract

In this work, the terahertz time-domain spectroscopy method analyzed solutions of bovine serum albumin (BSA) in two high concentrations (50 and 334 mg/mL) at three pH values (2.5, 6.5, 8.5) and the same solvents without protein, at 25°C. The spectra of dry BSA were also recorded. For the first time, a method for determining the complex dielectric permittivity of protein molecules in aqueous solutions, without the dielectric contribution of the aqueous phase, is proposed. It is shown that the dielectric permittivity of dissolved and dry BSA (lyophilized, in the native conformation) differ significantly in the terahertz frequency range. These differences are small near 70 cm^−1^, but they increase greatly with decreasing frequency. It was found that the dielectric losses of protein molecules in solution are close to the dielectric losses of the aqueous environment, which in this frequency range are determined by intermolecular relaxation processes of water. Since dielectric losses are directly related to molecular dynamics, this fact shows that the intramolecular dynamics of the protein completely adjusts to the intermolecular dynamics of the aqueous environment. It also indicates that the native conformation does not determine all the fundamental characteristics of a protein molecule, in particular, it does not determine the dynamics of the protein, which significantly depends on the water environment.

## 1 Introduction

Biomolecules are the molecular basis of life. But they achieve functional states only in an aqueous environment. The most extensive class of biomolecules is proteins, the native conformation of which is formed in water with the participation of hydrophobic effects (Privalov and Gill, 1988). But not only does water affect the structure of protein, protein also affects the structure of water, forming a hydrate shell (Penkov, 2023). In other words, hydrated protein is a single mutually consistent system. For a long time it was believed that the hydrate shell consists of a small number of strongly bound water molecules in one or two hydrate layers. However, in the 2000s, terahertz (THz) spectroscopy showed that the hydrate shells of proteins are much more extended—up to several nanometers (Ebbinghaus et al., 2007; Born et al., 2009; Heyden et al., 2012; Bye et al., 2014; Novelli et al., 2017; Wang et al., 2019). The more general term “dynamic hydrate shells” has emerged (Ebbinghaus et al., 2007; Born et al., 2009; Heyden et al., 2010; Conti Nibali and Havenith, 2014), which include not only the nearest strongly bound water molecules, but also more distant hydration regions with altered intermolecular dynamics. In a series of our works, the dynamic hydrate shells of various biomolecules were studied based on the analysis of the complex dielectric permittivity of their solutions in the THz range (Penkov et al., 2018; Penkov, 2021; Penkov et al., 2021; Penkova et al., 2021; Penkov et al., 2022; Penkov, 2024). An important stage of these studies was the subtraction of the dielectric contribution of biomolecules from the permittivity of solutions. For this purpose, effective medium models were used, including the model we developed theoretically (Penkov N. and Penkova N. A., 2021). Based on THz spectra using molecular dynamics simulations, it was shown that the dynamics of the hydrate shell of a protein can correlate with the intrinsic dynamics of the protein (Born et al., 2009; Heyden et al., 2010; Conti Nibali and Havenith, 2014; Pezzotti et al., 2023). Thus, THz spectroscopy has demonstrated unsurpassed sensitivity in the analysis of molecular dynamics in aqueous solutions of biomolecules, mainly proteins.

In this paper, the following question is raised: are all the properties of a protein molecule determined by its conformation? Or, besides structural characteristics, are there dynamic characteristics that are activated by the aqueous environment? The study of protein dynamics has been paid attention in many studies using various experimental techniques (Krupianskii et al., 1987; Johansson and Lindorff-Larsen, 2018; Schirò and Weik, 2019; Campitelli et al., 2020; Hodge et al., 2020; Bondar, 2022; Stingaciu, 2022). However, these studies completely ignored the possibilities of dielectric spectroscopy.

Molecular dynamics can be analyzed using complex dielectric spectra. Each intramolecular or intermolecular degree of freedom is reflected in the dielectric permittivity at characteristic frequencies. The intramolecular dynamics of macromolecules, such as proteins, is well manifested in the THz frequency range (Markelz, 2008; Wei et al., 2018). Thus, it is possible to try to answer the above question by comparing the THz dielectric spectra of the protein in dry and hydrated form. When obtaining dry protein, it is important to avoid significant changes in its structure, for example, denaturation. For small model proteins such as bovine serum albumin (BSA), this structure can be preserved by lyophilization. And measuring the spectrum of dry protein is not difficult. The analysis of hydrated protein is much more complicated. Since the dynamic hydrate shell of a protein molecule can exceed its own volume (Leitner et al., 2008; Heyden et al., 2012; Wallace et al., 2015; Wang et al., 2019), a protein can be considered fully hydrated only in an aqueous solution. Therefore, it is necessary to find a way to isolate the dielectric spectrum of a protein from the spectra of its solutions. In this paper, this problem is solved using an effective medium model that interconnects THz dielectric spectra of protein solutions of two concentrations, pure solvent and dissolved protein.

## 2 Methods

### 2.1 Preparation of samples

Solutions of BSA (>99%, Dia-M, Russia) were prepared in two concentrations: 334 and 50 mg/mL. Aqueous solutions of 150 mm NaCl at three pH values: 2.5, 6.5, 8.5 were used as solvents for protein. In total, six types of BSA solutions and three types of pure solvents were analyzed. The solvents were prepared from water MilliQ (Millipore, Germany) and NaCl (Sigma-Aldrich, United States), and the pH was set using HCl (Sigma-Aldrich, United States) and NaOH (Sigma-Aldrich, United States) additives. The protein was dissolved at room temperature with shaking on Bio Vortex V1 (Biosan, Latvia) for 15 min. During this time, the protein clots visible to the eye completely disappeared in a solution with a concentration of 334 mg/mL. After that, the solutions were degassed in a degassing station (TA Instruments, New Castle, United States) for 15 min to eliminate air bubbles that may interfere with spectral measurements (Section 2.2).

Also, dry protein samples were prepared for spectral measurements by mixing 15 mg of BSA with 150 mg of polyethylene powder (Sigma-Aldrich, United States). The mixture was evenly distributed by shaking in a test tube. No additional grinding was carried out. Since the protein is quite sticky, during the grinding process it would stick to the walls of a mortar or ball mill. This would lead to an uncontrolled loss of protein mass in the sample. A mixture of protein and polyethylene was pressed into pellets with a diameter of 13 mm at a pressure of 0.75 kbar.

Preparation of pellets is usually carried out under greater pressure to reduce the looseness of the pellets and minimize the radiation scattering in the air cavities. However, at greater pressures there is a risk of a baric change in the structure of the protein (Ceolın, 2000). During spectral measurements (Section 2.2), the possible looseness of the pellets with BSA was compensated by the similar looseness of the background pellets, which were prepared from 150 mg of pure polyethylene compressed under the same pressure. In addition, it was verified that the difference in the thickness of pellets compressed at a pressure of 0.75 and 4 kbar (typical pressure used for spectral studies of dry organic matter) does not exceed 0.25%. That is, under a pressure of 0.75 kbar, we received almost extremely dense pellets due to the softness of its components.

### 2.2 Obtaining complex dielectric spectra in the THz range

The terahertz time-domain spectroscopy (THz-TDS) method was used. The spectra were measured on a TPS Spectra 3000 spectrometer (Teraview, United Kingdom) in the range of 10–110 cm^−1^ with a spectral resolution of 4 cm^−1^. This method implements a procedure for coherent generation and detection of picosecond electromagnetic pulses. Picosecond pulses are characterized by a spectrum with a width of several THz, measured from the zero frequency, which allows spectral measurements to be carried out in the THz region. Without going into the details of the THz-TDS method, which can be found in the literature (Lee, 2009), it allows simultaneous measurement of the absorption spectrum $\alpha \left(\nu \right)$ and the spectrum of the refractive index $n\left(\nu \right)$ of a studied sample. From these spectra, the real $\varepsilon^{\prime }\left(\nu \right)$ and the imaginary $\varepsilon^{\prime \prime }\left(\nu \right)$ parts of the dielectric permittivity spectrum are calculated using standard ratios:

$$\varepsilon^{\prime }\left(\nu \right)=n^{2}\left(\nu \right)-{\left[\frac{\alpha \left(\nu \right)}{4\pi \nu }\right]}^{2},\;\varepsilon^{\prime \prime }\left(\nu \right)=\frac{n\left(\nu \right)\alpha \left(\nu \right)}{2\pi \nu }$$ (1)

where ν is the wavenumber measured in cm^−1^. The absorption spectrum is related to the transmission spectrum $Tr\left(\nu \right)$ through the ratio $\alpha \left(\nu \right)=-\ln\left(Tr\left(\nu \right)\right)/d$ , where *d* is the thickness of the sample.

To obtain the spectra of dry protein, the spectra of a pellet with BSA and the background spectra of pure polyethylene pellet with a mass equal to the mass of polyethylene in pellet with BSA were recorded. To obtain the spectra of $\alpha \left(\nu \right)$ , $n\left(\nu \right)$ and then calculate the dielectric spectra, it is necessary to set the thickness *d* of the protein layer in the pellet, which was calculated based on the protein mass *m*, the pellet area *S* and the density of BSA ρ using Eq. 2:

$$d=\frac{m}{\rho \;S}$$ (2)

The protein weight was 15 mg, the pellet area with a diameter of 1.3 cm was 1.33 cm^2^, and the BSA density was about 1.41 g/cm^3^ (Fischer et al., 2009). As a result, the effective thickness of the protein in the pellet was 80 µm.

Two cuvettes with windows made of Z-cut quartz were used to record the spectra of solutions. Teflon spacers with thicknesses of 50 and 100 μm were installed between the windows of these cuvettes. Since the distance between the cuvette windows may differ slightly from the thickness of the spacer, the exact distances were measured interferometrically on empty assembled cuvettes. For this purpose, a Fourier-transform infrared spectrometer Nicolet 6,700 (Thermo, United States) was used in the near infrared range, where Z-cut quartz is transparent. The technique of measuring the thickness of transparent layers using etalon effect is well known and has been used by us before (Penkov et al., 2018). The exact distances between the windows in the two cuvettes were 100.56 and 50.12 μm.

The spectrum of each solution was recorded in two specified cuvettes. The spectrum of the solution in the cuvette with larger thickness was considered the “spectrum of the sample,” and the spectrum of the same solution in the cuvette with smaller thickness was considered the “background spectrum.” Thus, the final spectra of absorption and refractive index were determined for a solution layer with a difference thickness of 50.48 μm with a complete subtraction of the contribution of the cuvette windows. This approach allows us to obtain much more accurate spectra than with the standard recording of the spectrum of an empty cell as a background. This eliminates distortions of the spectra associated with the etalon effect, as well as with the difference in the reflection coefficient of radiation from an empty and filled cuvette (Penkov et al., 2015). From the obtained solutions spectra of $\alpha \left(\nu \right)$ and $n\left(\nu \right)$ , the dielectric spectra were calculated using Eq. 1.

All spectral measurements of the solutions were carried out in cuvettes placed in a thermostatic holder at a temperature of 25 C ± 0.2 C. The optical part of the spectrometer was purged with dried air with a steadily reduced water vapor content by more than 20 times relative to laboratory air. After installing the sample cuvette in the cuvette compartment, a pause of 5 min was maintained to stabilize the sample temperature and purge the spectrometer.

The spectra of each sample were measured at least 30 times for the possibility of averaging and statistical analysis.

### 2.3 Calculation of the complex dielectric permittivity spectra of a protein

Obtaining dielectric spectra of dry protein can be easily performed according to the procedure described in Section 2.2. However, determining the dielectric spectra of protein in solution is much more difficult, which is the main task of this study. From the point of view of the electrodynamics of continuous media (Landau et al., 1984), a protein solution can be considered a two-phase system consisting of a continuous aqueous medium with inclusions of protein molecules. In some cases, it is desirable to highlight the third phase of hydrate shells.

Taking into account the molecular weight of BSA of 66.5 kDa and the protein density of 1.41 g/mL, in the approximation of the sphericity of a protein globule, its diameter is 5.3 nm. On the totality of available scientific evidence we can conclude that the dynamic hydration shell of the protein has a thickness of about 1.5–2 nm (Ebbinghaus et al., 2007; Born et al., 2009; Heyden et al., 2012; Bye et al., 2014; Wang et al., 2019). Based on this, the volume of hydrated water exceeds the volume of protein by about 3–4 times. The volume fraction of BSA in a solution with a concentration of 334 mg/mL is $f_{p}=0.237$ , therefore, almost all the water in this solution belongs to dynamic hydrate shells. These hydrate shells are, of course, heterogeneous, and their characteristics depend on the distance to the surface of the protein molecule. However, it is essential that in a solution with a BSA concentration of 334 mg/mL, almost all the water is in the area of significant influence of the protein and can be considered hydrated. Such a solution as a two-phase system can be described using a well-known and simple effective medium model of Bruggeman (Bruggeman, 1935):

$$f_{p}\frac{\varepsilon_{p}^{*}-\varepsilon_{cs}^{*}}{\varepsilon_{p}^{*}+2\varepsilon_{cs}^{*}}+\left(1-f_{p}\right)\frac{\varepsilon_{sh}^{*}-\varepsilon_{cs}^{*}}{\varepsilon_{sh}^{*}+2\varepsilon_{cs}^{*}}=0$$ (3)

where $\varepsilon_{cs}^{*}$ , $\varepsilon_{p}^{*}$ are the dielectric permittivity of the concentrated solution (334 mg/mL) and protein molecules, respectively, and $\varepsilon_{sh}^{*}$ is the average dielectric permittivity of the aqueous phase of this solution, which entirely belongs to dynamic hydrate shells.

Note that there are various effective medium models, except for the Bruggeman model. However, this model has proven itself well in the analysis of two-phase systems not only with spherical inclusions, but also with randomly arranged inclusions of arbitrary shape (Choy, 1999). At the same time, the Bruggeman model does not require setting additional unknown parameters of the inclusions shape. To describe the dielectric response of heterogeneous systems in alternating fields, the Bruggeman model is applicable under the following two conditions: small inclusions compared to the wavelength of radiation and weak absorption of radiation by individual inclusions (Sihvola, 1999). Obviously, both of these conditions are fulfilled when analyzing solutions of globular proteins in the terahertz range.

From Eq. 3, $\varepsilon_{sh}^{*}$ can be expressed as follows:

$$\varepsilon_{sh}^{*}=\frac{\left(1-3f_{p}\right)\varepsilon_{p}^{*}\varepsilon_{cs}^{*}+2\varepsilon_{cs}^{{*}^{2}}}{\varepsilon_{p}^{*}+\left(2-3f_{p}\right)\varepsilon_{cs}^{*}}$$ (4)

A less concentrated BSA solution (50 mg/mL) can be considered as a three-phase dielectric system consisting of protein inclusions, hydrate shells and the rest of the aqueous phase of the solution, identical to the pure solvent. For a three-phase system, the Bruggeman model is written as follows:

$$f\frac{\varepsilon_{p}^{*}-\varepsilon_{s}^{*}}{\varepsilon_{p}^{*}+2\varepsilon_{s}^{*}}+f_{sh}\frac{\varepsilon_{sh}^{*}-\varepsilon_{s}^{*}}{\varepsilon_{sh}^{*}+2\varepsilon_{s}^{*}}+\left(1-f-f_{sh}\right)\frac{\varepsilon_{w}^{*}-\varepsilon_{s}^{*}}{\varepsilon_{w}^{*}+2\varepsilon_{s}^{*}}=0$$ (5)

where f and $f_{sh}$ are the volume fractions of the protein and its hydrate shells in a BSA solution with a concentration of 50 mg/mL, $\varepsilon_{s}^{*}$ is the dielectric permittivity of this solution, and $\varepsilon_{w}^{*}$ is the dielectric permittivity of the aqueous phase of the solution located outside the hydrate shells.

It is obvious that the dynamic hydrate shell does not have a clear outer boundary. In fact, the effect of protein on the structural and dynamic characteristics of water decreases with increasing distance from the protein surface. Therefore, the transition from hydrated to non-hydrated water is somewhat conditional. However, since we analyze the system using THz spectroscopy, which is sensitive to changes in water under the influence of protein at distances up to 1.5–2 nm, at longer distances this method will already detect undisturbed water identical to water in a pure solvent.

According to the above estimates, the dynamic hydrate shells of the protein approximately correspond to the total volume of water in a BSA solution with a concentration of 334 mg/mL. Then the volume fraction of hydrate shells $f_{sh}$ in a solution with a BSA concentration of 50 mg/mL can be calculated from the volume fraction of the aqueous phase in a solution of 334 mg/mL multiplied by the ratio of protein concentrations in these two solutions: $f_{sh}=\left(1-0.237\right)*50/334=0.114$ . The volume fraction of the protein in this case is f=0.0355 .

After substituting the expression (Eq. 4) for $\varepsilon_{sh}^{*}$ into Eq. 5, we have the following equation:

$$f\frac{\varepsilon_{p}^{*}-\varepsilon_{s}^{*}}{\varepsilon_{p}^{*}+2\varepsilon_{s}^{*}}+f_{sh}\frac{\frac{\left(1-3f_{p}\right)\varepsilon_{p}^{*}\varepsilon_{cs}^{*}+2\varepsilon_{cs}^{{*}^{2}}}{\varepsilon_{p}^{*}+\left(2-3f_{p}\right)\varepsilon_{cs}^{*}}-\varepsilon_{s}^{*}}{\frac{\left(1-3f_{p}\right)\varepsilon_{p}^{*}\varepsilon_{cs}^{*}+2\varepsilon_{cs}^{{*}^{2}}}{\varepsilon_{p}^{*}+\left(2-3f_{p}\right)\varepsilon_{cs}^{*}}+2\varepsilon_{s}^{*}}+\left(1-f-f_{sh}\right)\frac{\varepsilon_{w}^{*}-\varepsilon_{s}^{*}}{\varepsilon_{w}^{*}+2\varepsilon_{s}^{*}}=0$$ (6)

The coefficients f , $f_{sh}$ and $f_{p}$ are known, the spectra $\varepsilon_{w}^{*}$ , $\varepsilon_{s}^{*}$ and $\varepsilon_{cs}^{*}$ are obtained experimentally, therefore Eq. 6 contains one unknown complex variable $\varepsilon_{p}^{*}$ . The solution of Eq. 6 allows us to find $\varepsilon_{p}^{*}$ at any frequency for which the permittivities $\varepsilon_{w}^{*}$ , $\varepsilon_{s}^{*}$ and $\varepsilon_{cs}^{*}$ are given. Thus, this makes it possible to calculate the complex dielectric spectrum of the protein $\varepsilon_{p}^{*}$ in the solution, from which the dielectric contribution of the aqueous phase is completely excluded.

## 3 Results and discussion

The analytical solution of Eq. 6 gives an extremely cumbersome expression, which is not given here. Due to the large number of coefficients in large degrees, the solution of this equation turns out to be very sensitive to the parameters included in it. To obtain stable values of $\varepsilon_{p}^{*}$ , it is necessary to set the parameters $\varepsilon_{s}^{*}$ , $\varepsilon_{cs}^{*}$ and $\varepsilon_{w}^{*}$ with an accuracy higher than 0.001. Deviations of these parameters by the values of the real spread of experimental data (about 0.01) lead to a change in the calculated $\varepsilon_{p}^{*}$ several times, which cannot be considered acceptable. Therefore, it was necessary to look for ways to approximate, but more sustainable solution.

**FIGURE 1:**
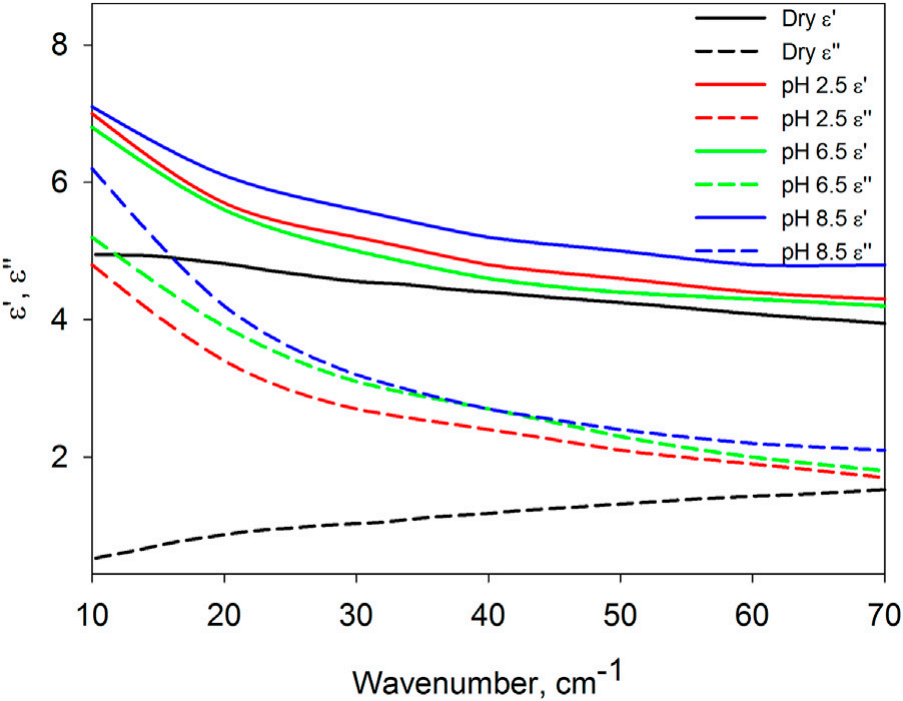
Averaged dielectric spectra, their real ( $\varepsilon^{\prime }$ ) and imaginary ( $\varepsilon^{\prime \prime }$ ) parts, of dry BSA and BSA in solutions with pH = 2.5, 6.5, 8.5.

The analysis of Eq. 6 shows that the first term is small compared to the other two terms due to the small coefficient *f*. If this term is neglected, the degree of the equation decreases, and the solution becomes much more stable, so that the experimental variation of the parameters included in Eq. 6 does not significantly affect the determined values of $\varepsilon_{p}^{*}$ . Eq. 6 was solved in the specified approximation by setting the experimental values of $\varepsilon_{s}^{*}$ , $\varepsilon_{cs}^{*}$ and $\varepsilon_{w}^{*}$ at different frequencies. At the same time, after finding $\varepsilon_{p}^{*}$ for each frequency, it was checked that the second and third terms of Eq. 6 significantly (by about an order of magnitude) exceed the first term, that is, the correctness of neglecting it is respected. This condition is fulfilled for spectra in the range from 10 to 70 cm^−1^, which are shown in Figure 1.

Figure 1 shows that the dielectric spectra of protein in solution are strikingly different from those of dry protein. The differences between the real and imaginary parts of the dielectric permittivity are small near 70 cm^−1^, but increase sharply with decreasing frequency. Despite the approximate nature of the spectra found, the opposite frequency dependence of the dielectric losses ( $\varepsilon^{\prime \prime }$ ) of dry and dissolved protein attracts special attention. An increase in dielectric losses with a decrease in frequency indicates that the protein in an aqueous environment exhibits pronounced intramolecular dynamics at frequencies less than 2 THz, which strongly attenuates in dehydrated protein.

**FIGURE 2:**
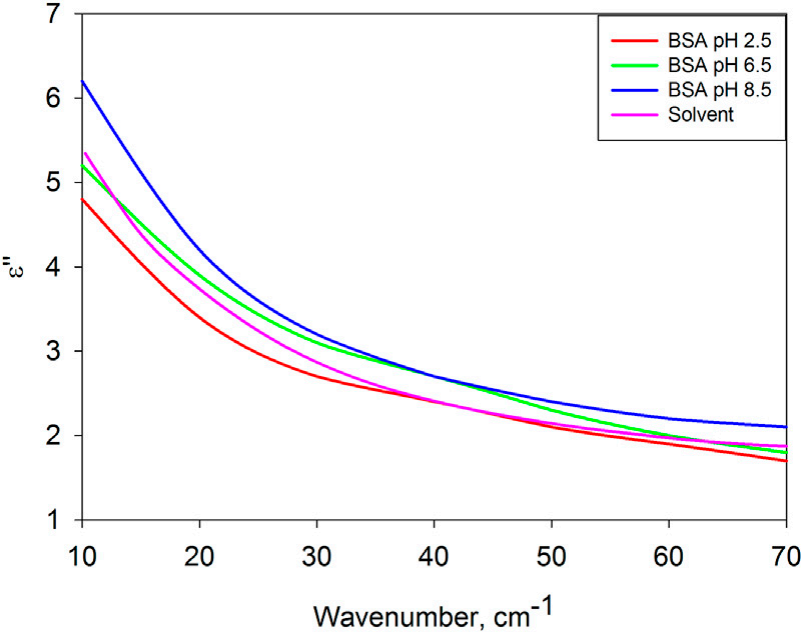
Dielectric losses of BSA in solutions with pH = 2.5, 6.5, 8.5 and solvent at pH 6.5 (solvent spectra at pH = 2.5 and 8.5 are practically indistinguishable from the case of pH = 6.5, therefore they are not given).

Figure 2 compares the dielectric losses of a dissolved protein with a solvent. Surprisingly, they are close to each other in the entire frequency range.

**FIGURE 3:**
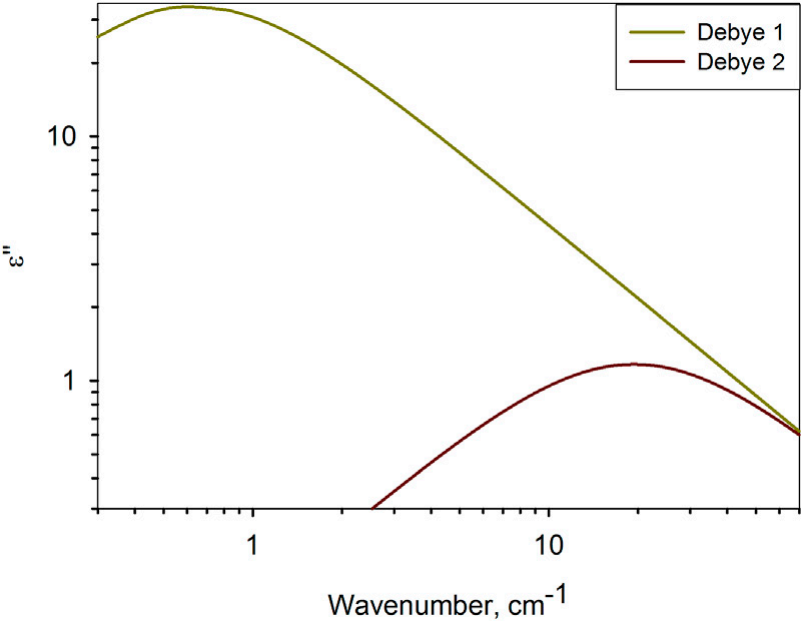
Relaxation bands of water at 25°C (Penkov N. V. and Penkova N., 2021), determining the dielectric response of water in the range of 10–70 cm^−1^. Debye 1 describes Debye relaxation (von Hippel, 1988a; von Hippel, 1988b), and Debye 2 describes high–frequency relaxation of water (Yada et al., 2008; Penkov et al., 2013).

According to the dielectric characteristics, the solvents used are almost identical to water. The dielectric spectra of water in the frequency range under consideration are determined by two relaxation bands (Figure 3) due to intermolecular dynamics of bound (von Hippel, 1988a; von Hippel, 1988b; Barthel et al., 1990; Lyashchenko and Lileev, 2010) and free (Barthel et al., 1990; Yada et al., 2008; Penkov et al., 2013; Shiraga et al., 2017) water molecules. This type of molecular organization of liquid water has no common features with the protein globule. However, their dielectric response is almost the same. It is generally believed that a biomolecule, by binding water on its surface, completely determines the structure of its hydrate shell. The obtained result gives grounds to assert the opposite, but for molecular dynamics: in solution, the intramolecular dynamics of a protein molecule is slaved to the intermolecular dynamics of the aqueous environment.

In this work, three pH values of the solutions were selected, at which BSA takes on different conformations (Rosenoer, 1977; Cao et al., 2013): at pH 2.5, the protein has an extended form, which is formed as a result of the destruction of the inner helical part of domain I; at pH 6.5, protein is in native form; at pH 8.5, the protein passes into the basic form, in which there is also a change in the domain I. It is important that with all these conformations, the protein retains colloidal stability, does not denature and does not aggregate (Penkov et al., 2018; Penkov, 2024). Previously, we showed that the structural and dynamic characteristics of hydrate shells depend on the conformation of biomolecules (Penkov, 2021; Penkov et al., 2021; Penkova et al., 2021; Penkov et al., 2022), including proteins (Penkov et al., 2018). Based on this, it can be expected that the dynamics of the protein itself depends on its conformation. Unfortunately, the data presented in Figure 1, taking into account the proximity of the curves and the approximation of the equation solution indicated above, do not allow us to establish reliable differences in the dielectric characteristics of the protein in different conformations.

Nevertheless, the study of the conformational specificity of protein dynamics is of deep scientific interest. As it has been shown in various examples (Born et al., 2009; Ebbinghaus et al., 2012; Meister et al., 2013; Adams et al., 2021), mutations leading to changes in the rigidness of a protein molecule affect the ability of proteins to perform their biological functions. Conformational changes also lead to a change in the rigidness of the protein molecule and clearly affect the functional abilities of the protein. Any mechanical changes must be accompanied by a change in dynamic characteristics. The determination of the dependence of the protein dynamics in solution on its conformation may be of great importance for understanding the fundamental foundations of biochemistry and molecular biology, in particular the principles of enzyme functioning (Palmer and Bonner, 2007). However, to study this, it is necessary either to learn how to obtain much more accurate dielectric spectra using the THz-TDS method, or to look for other ways to determine the dynamic characteristics of proteins in solutions.

Nevertheless, in this work, in terms of dielectric permittivity, significant differences in the dynamics of BSA in dissolved and dry form are clearly shown, including for BSA of the same native conformation. Using the example of a model protein (BSA) this shows that the native conformation itself does not determine all the fundamental characteristics of protein molecules, in particular, it does not determine their dynamics. But the aqueous environment has a key influence on protein dynamics. This fact, when examined in more detail, can have far-reaching consequences for physical chemistry, biochemistry and molecular biophysics.

## Data Availability

The raw data supporting the conclusions of this article will be made available by the author, without undue reservation.
